# Establishment of an experimental model for MHC homo-to-hetero transplantation

**DOI:** 10.1038/s41598-020-69784-4

**Published:** 2020-08-11

**Authors:** Tomoki Murata, Haruka Wada, Ryo Otsuka, Airi Sasaki, Hyuma Tsuji, Mizuho Itoh, Nanami Eguchi, Tatsuo Kawai, Ken-ichiro Seino

**Affiliations:** 1 Division of Immunobiology, Institute for Genetic Medicine, Hokkaido University, Kita-15, Nishi-7, Kita-ku, Sapporo, Hokkaido 060-0815 Japan; 2 Department of Transplant Surgery, Massachusetts General Hospital, Boston, MA USA

**Keywords:** Allotransplantation, T cells, Induced pluripotent stem cells, MHC

## Abstract

Preventing rejection is a major challenge in transplantation medicine, even when using pluripotent stem cell-derived grafts. In iPS cell (iPSC)-based transplantation, to reduce the risk of rejection, it is thought to be optimal that preparing the cells from donors whose human leukocyte antigen-haplotype are homozygous. Generally, this approach is referred to as major histocompatibility complex (MHC) homo-to-hetero transplantation, which is MHC-matched but minor antigen-mismatched. To investigate the immune response in the MHC homo-to-hetero transplantation, we established a murine experimental system in which MHC-matched but minor antigen-mismatched tissue (skin) grafts were transplanted into MHC-heterozygous recipients. Unexpectedly, only minor antigen-mismatched grafts were rejected at the same time points as rejection of fully allogeneic grafts. A vigorous anti-donor type T cell response was detected in vitro and conventional immunosuppressants targeting T cell activation had limited effects on controlling rejection. However, anti-donor antibodies were not detected only in the minor antigen-mismatched transplantation. This murine transplantation model can be used to further analyze immunological subjects for MHC homo-to-hetero iPSC-based transplantation.

## Introduction

In transplantation medicine, including organ or tissue transplantation, promoting graft acceptance and prolonging graft survival are important issues. Rejection is the major cause of graft loss, and many studies focusing on preventing rejection have been performed. Although the major cause of graft loss is human leukocyte antigen (HLA) (major histocompatibility complex, MHC) mismatches, in addition to this, other antigens, such as minor antigens, can also cause rejection^1,2^. Therefore, allogeneic recipients are usually administered immunosuppressants throughout their lives to control rejection.

Pluripotent stem cells (PSCs) used for cell or tissue transplantation are a subject suffered from rejection when transplanted in allogeneic recipients^3,4^. Using autologous iPSCs is an ideal approach for avoiding rejection^3^. However, preparation of these cells is time-consuming and costly. In contrast, stocking of “off-the-shelf” allogeneic iPSCs has been proposed^3,4^. To reduce the risk of rejection in “off-the-shelf” iPS-derived cell or tissue transplantation, MHC (HLA)-homozygous iPSCs stock project has been suggested^3,4^. In preclinical studies, monkey dopaminergic neurons or cardiomyocytes derived from MHC homozygous iPSCs were transplanted into MHC heterozygous recipients^5,6^. In this type of MHC homo-to-hetero transplantation, although the immune response was decreased, administration of immunosuppressants was still required to prevent rejection. The immune responses in this case are considered to be induced by mismatches of minor antigens. However, the immunological features of the MHC-matched but minor antigen-mismatched transplantation are not well-understood.

In fact, MHC-matching is advantageous for reducing the risk of rejection, including chronic antibody mediated rejection (CABMR), in clinical kidney transplantation^7^. However, there are few cases in which the types of MHCs between donors and recipients are completely matched^7^. This is because MHC molecules are highly polymorphic, and it is very difficult to find a completely suitable donor for each recipient. For these reasons, the immunological aspects of MHC-matched transplantation have not been fully elucidated. In this study, we firstly established an experimental mouse model in which MHC-matched but minor antigen-mismatched tissue (skin) was transplanted. The immunological characteristics of the transplantation have been examined in detail. The information from this study would be beneficial for developing iPSC-based transplantation.

## Results

### The survival of skin grafts depends on the combination of donors and recipients in MHC-matched but minor antigen-mismatched transplantation

**Figure 1:**
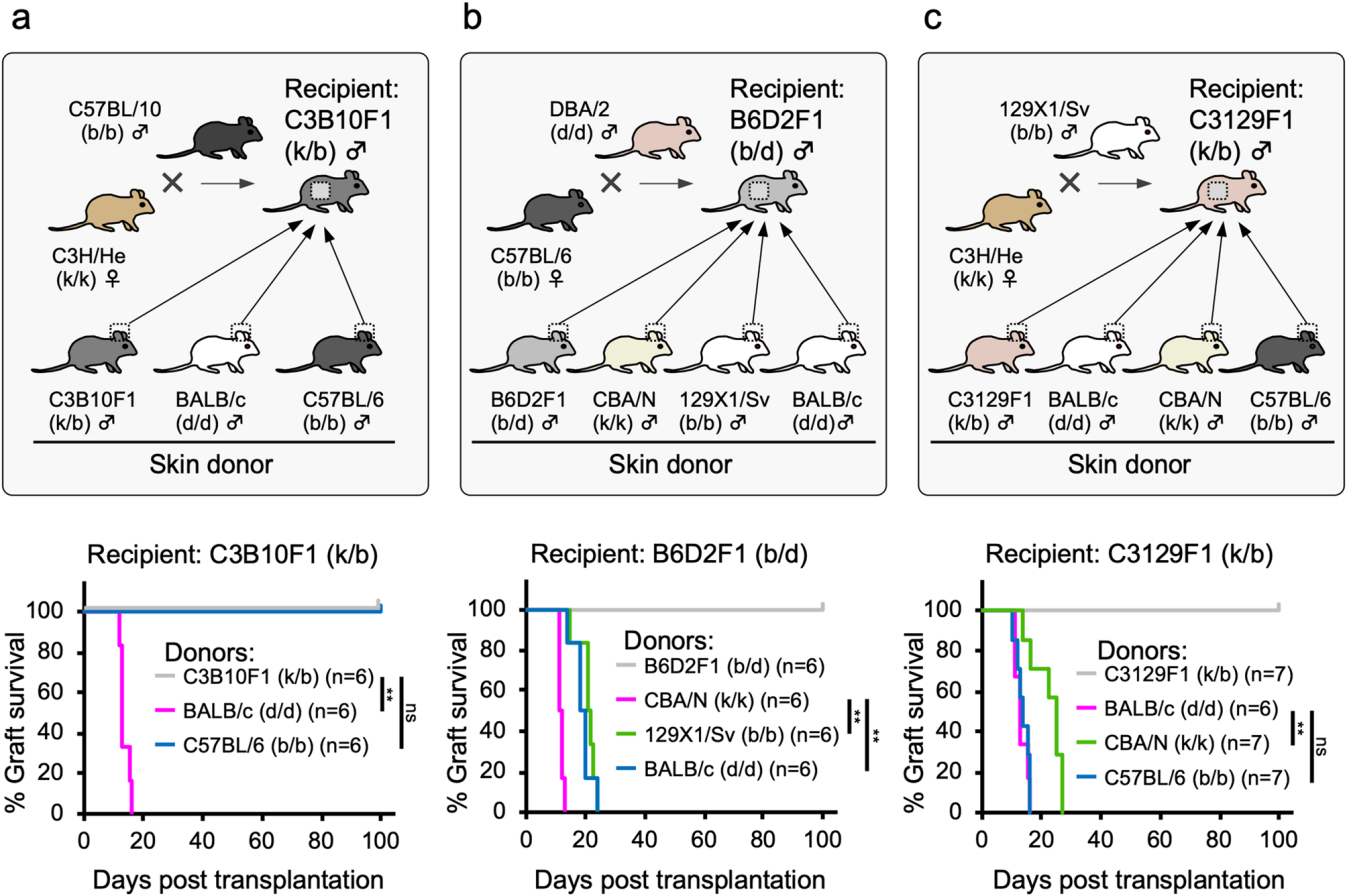
Combination between donor and recipient is critical for determining the extent of immune rejection in MHC-matched but minor antigen-mismatched transplantation. Auto or allogeneic skin grafts (BALB/c (MHC haplotype: d/d), C57BL/6 (b/b), CBA/N (k/k), or 129X1/Sv (b/b)) were transplanted into MHC-heterozygous recipients. The recipients were (**a**) C3B10F1 (k/b), (**b**) B6D2F1 (b/d), and (**c**) C3129F1 (k/b). **p < 0.01, log-rank test.

We first established a murine experimental model as follows: C3H/He × C57BL/10 (B10) F1 (C3B10F1, MHC haplotype: k/b), C57BL/6J (B6) × DBA/2 (B6D2F1: b/d), and C3H/He × 129X1/Sv F1 (C3129F1: k/b) mice as recipients and B6 (b/b), CBA/N (k/k), 129X1/Sv (b/b), and BALB/c (d/d) mice as donors (Fig. 1). It is known that the strength of rejection is different among the type of grafts. Usually, tissue or cellular grafts are suffered from severer rejection than organ grafts in allogeneic transplantation^8^. Therefore, we chose skin (tissue) transplantation as a model because it is thought that iPSC-derived grafts are composed mainly of cells or tissues rather than of organs^9^. In C3B10F1 recipients, fully allogeneic BALB/c skin grafts were rejected as early as day 16, but B6 and auto (C3B10F1) skin grafts, were accepted for over 100 days (Fig. 1a). This is presumably because the B6 and B10 mouse strains were both derived from C57BL mice (this will be discussed later). In B6D2F1 or C3129F1 recipients, the survival time of minor antigen-mismatched 129X1/Sv (Fig. 1b), BALB/c (Fig. 1b), or CBA/N grafts (Fig. 1c) were slightly prolonged but grafts were rejected by day 27 (Fig. 1b,c). However, unexpectedly, only minor antigen-mismatched B6 grafts transplanted into C3129F1 recipients were rejected as early as fully allogeneic BALB/c grafts (Fig. 1c). These results indicate that even in the MHC-matched setting, minor antigen mismatch between the donor and recipient is critical for determining the extent of immune rejection. B6 skin grafts on C3129F1 recipients were rejected earliest among minor antigen-mismatched grafts in this study. Therefore, we chose this pair of donors and recipients as the experimental model.

### Immune cells infiltrate into skin grafts in MHC-matched but minor antigen-mismatched transplantation

**Figure 2:**
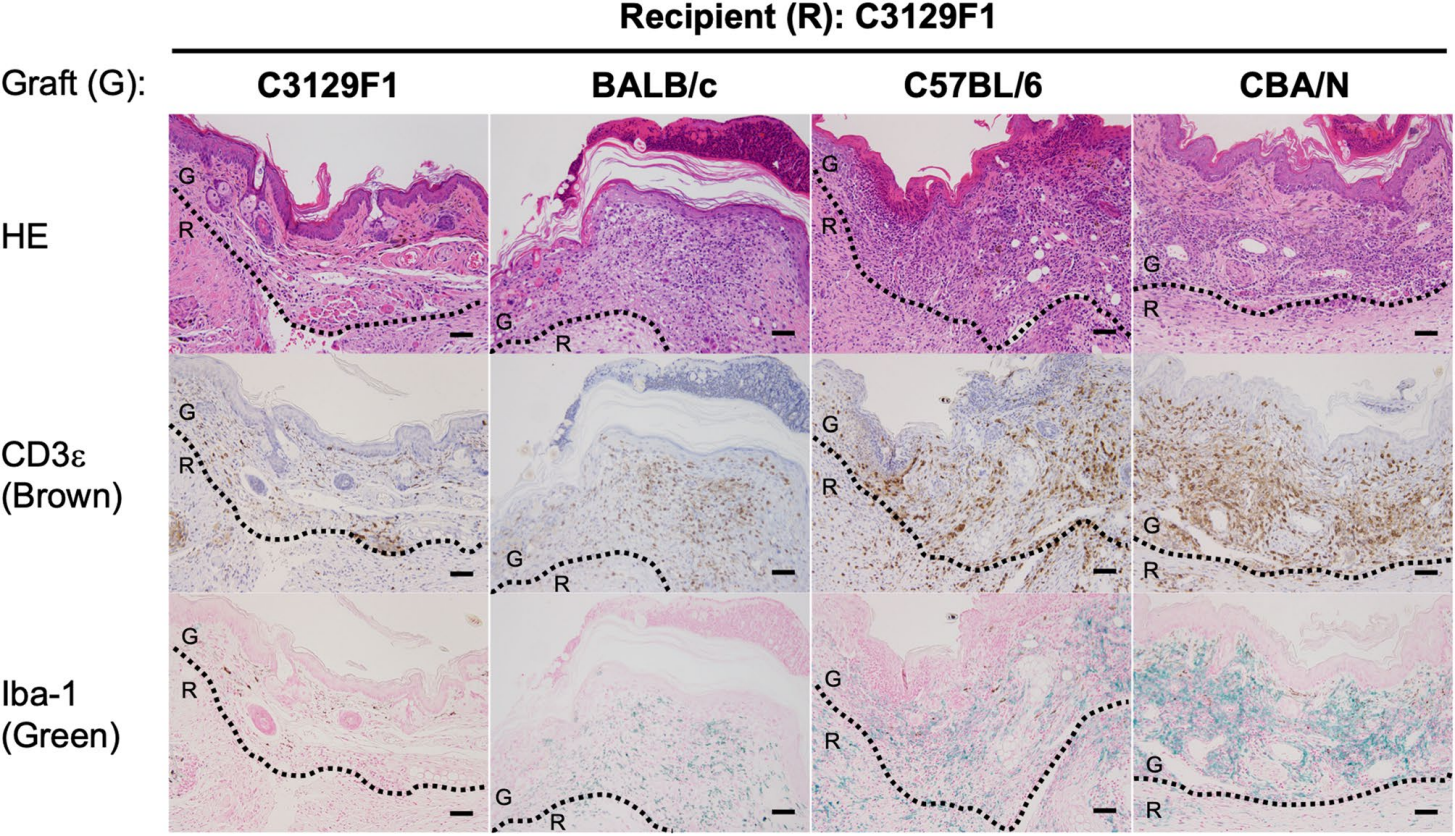
Severe infiltration of CD3^+^ or Iba-1^+^ cells in MHC-matched but minor antigen-mismatched skin grafts. At 7 days after transplantation and until 10 days after transplantation, the skin grafts were harvested and analyzed by immunohistochemistry. This is because BALB/c grafts were too severely destroyed to be analyzed at day 10. The broken lines indicate the border of the graft tissues (G) and recipient (R). Bars: 50 µm.

Next we examined infiltration of immune cells in the skin grafts to analyze immunological features of MHC homo-to-hetero transplantation. Immunohistochemical analyses revealed similar severe infiltration of CD3^+^ T and Iba-1^+^ cells in BALB/c, B6, and CBA/N grafts transplanted into C3129F1 recipients (Fig. 2), suggesting an active immune reaction at the site of transplantation. These results indicate that immune cells infiltrate into skin grafts and destruct them even if MHC is matched between donors and recipients.

### Anti-MHC matched but minor antigen-mismatched donor T cells are activated via indirect pathway in in vitro

**Figure 3:**
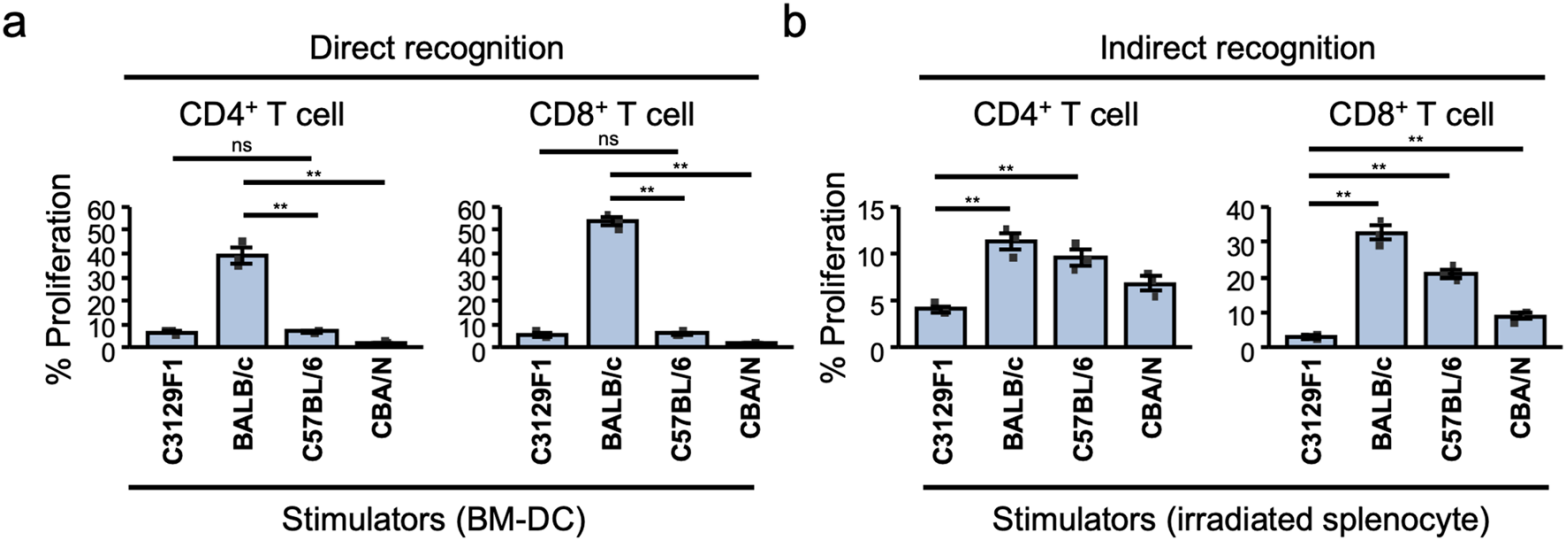
Recipient T cell response in MHC-matched but minor antigen-mismatched transplantation. (**a**) CFSE-labeled Thy1.2^+^ T cells isolated from naïve C3129F1 mice were co-cultured with bone marrow DCs derived from C3129F1, BALB/c, C57BL/6, or CBA/N mice. Four days later, the cells were stained with anti-CD4 or anti-CD8 antibodies, and then analyzed by flow cytometry. (**b**) To detect indirect alloantigen recognition, the CD19-negative fraction of splenocytes from C3129F1 recipient mice that had rejected skin grafts from BALB/c, C57BL/6, or CBA/N were used as responder cells. CFSE-labeled responder cells were co-cultured with 30 Gy irradiated splenocytes from C3129F1, BALB/c, C57BL/6, or CBA/N mice. Four days later, the cells were stained with anti-CD4 or anti-CD8 antibodies, and then analyzed by flow cytometry. For (**a**) and (**b**), T cell proliferation was evaluated based on CSFE reduction. Each bar shows the mean ± SD of triplicate cultures. The results are representative of three or two independent experiments. **p < 0.01, Tukey’s HSD test.

We then investigated T cell immune responses in MHC-matched but minor antigen-mismatched transplantation using the model of C3129F1 recipients. When C3129F1 CD4^+^ or CD8^+^ T cells were cocultured with dendritic cells (DCs) from donor mice, only T cells cocultured with fully allogeneic BALB/c DCs significantly responded (Fig. 3a). C3129F1 T cells cocultured with DCs derived from MHC-matched B6 or CBA/N mice did not respond (Fig. 3a), suggesting that direct pathway^1,10^ did not activate T cells in these cases. In contrast, to induce indirect pathway^1,10^, we cocultured splenocytes of C3129F1 and donors and found that the T cells clearly responded similarly in the extent of graft rejection (Fig. 3b). These results suggest that anti-donor T cells were activated via indirect pathway and involved in rejection in MHC-matched but minor antigen-mismatched (MHC homo-to-hetero) transplantation.

### Conventional immunosuppressants have limited effects on controlling rejection in MHC matched but minor antigen-mismatched skin transplantation

**Figure 4:**
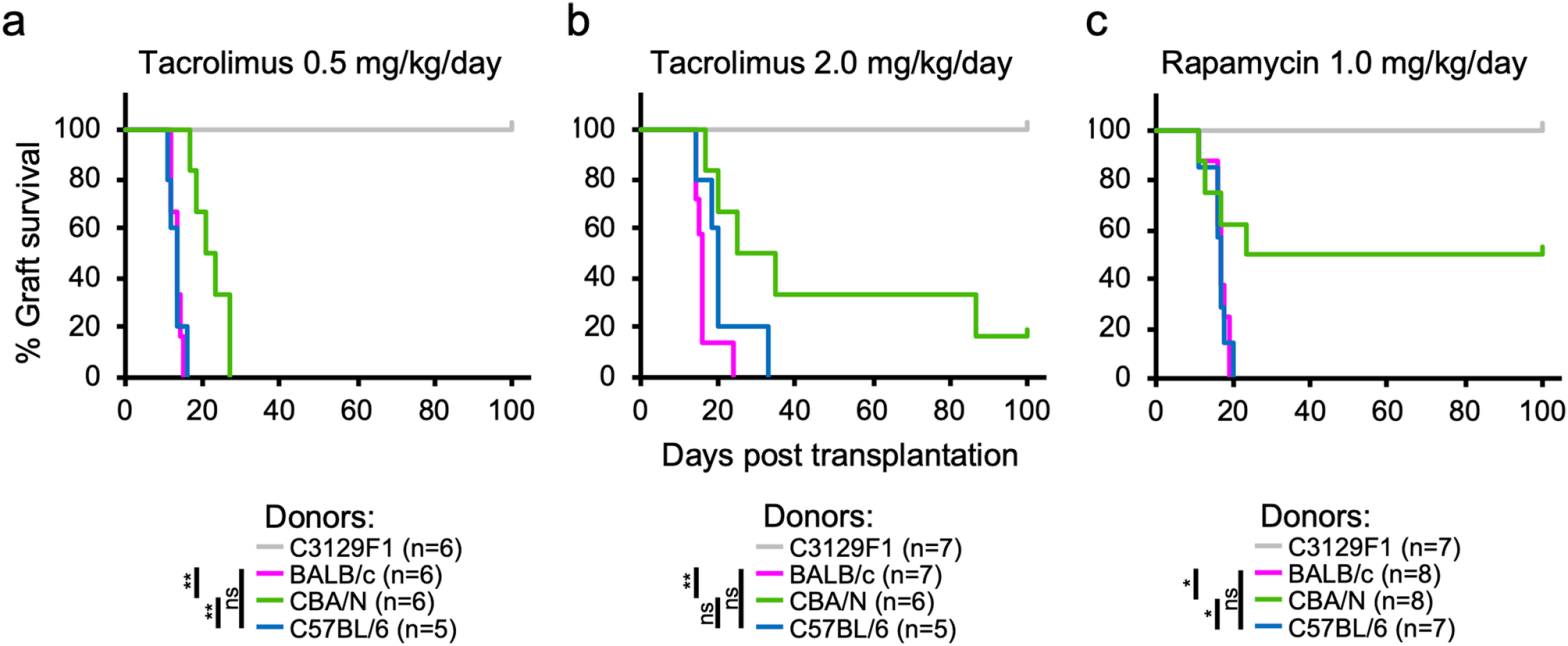
Limited effect of conventional immunosuppressants to control the rejection in MHC-matched but minor antigen-mismatched transplantation. C3129F1 recipient mice were intraperitoneally administered 0.5 mg/kg/day (**a**) or 2.0 mg/kg/day (**b**) tacrolimus, or 1.0 mg/kg/day rapamycin (**c**). The treatment was started from the day of skin transplantation. *p < 0.05, **p < 0.01, the log-rank test.

We next attempted to control graft rejection using conventional immunosuppressants that inhibit T cell activation. Daily treatment with 0.5 mg/kg tacrolimus did not result in long-term graft survival (Fig. 4a). Then, we increased the dose of tacrolimus to 2.0 mg/kg/day, which is more than 30-fold the maintenance dose used in human kidney transplantation^11^. This treatment significantly prolonged the survival of CBA/N skin grafts, but 80% of grafts were rejected by 100 days (Fig. 4b). In contrast, all B6 grafts were rejected as early as BALB/c grafts (Fig. 4b). When the recipients were treated with 1.0 mg/kg rapamycin daily, which is more than tenfold the maintenance dose used in human kidney transplantation^12^, the survival of CBA/N grafts was significantly prolonged, whereas there was no effect on B6 grafts (Fig. 4c). Therefore, in MHC-matched but minor antigen-mismatched transplantation, there may be similar cases in which conventional immunosuppressants cannot control the rejection.

### Anti-donor antibodies production is prevented in MHC matched but minor antigen-mismatched transplantation

**Figure 5:**
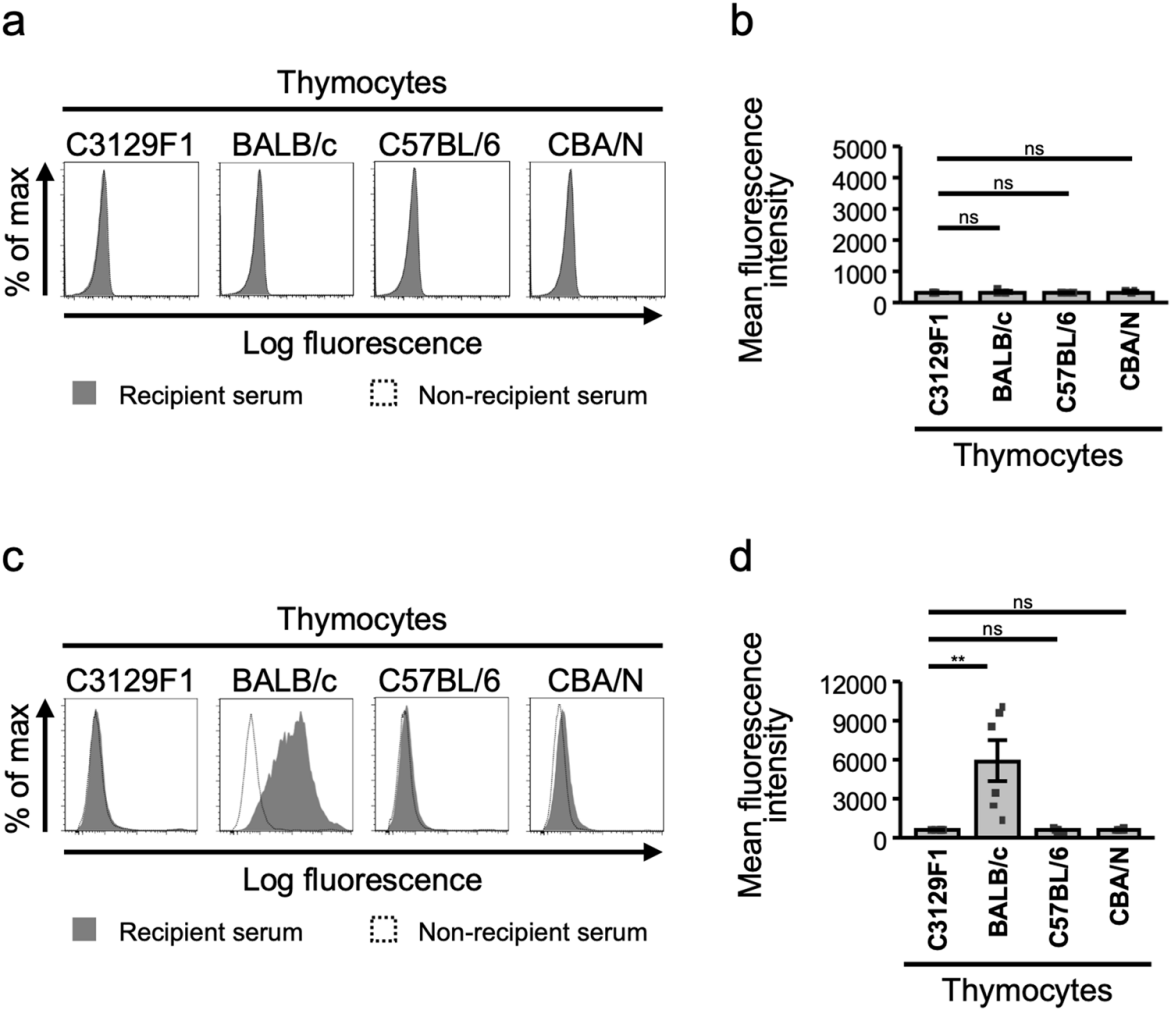
De novo anti-donor antibody production in MHC heterozygous recipients. (**a**–**d**) Sera were collected from recipient mice that underwent graft rejection. Thymocytes from the indicated mice were first stained with collected sera, and then further stained with PE-conjugated anti-mouse IgM antibody (**a**,**b**) or Alexa Fluor 488-conjugated anti-mouse IgG antibody (**c**,**d**). Thymocytes were analyzed by flow cytometry. The open histogram shows naïve C3129F1 mice sera staining, and filled histogram shows recipient C3129F1 sera staining. The data shown in (**b**,**d**) are representative of staining of the sera from six independent mice. Each bar shows the average mean fluorescence intensity ± SEM. **p < 0.01, Tukey’s HSD test.

In allogeneic transplantation, in addition to cellular immunity, anti-donor IgM or IgG antibodies can be produced and cause rejection^13^. Thus, we examined anti-donor antibody production in the skin transplantation model. Recipients’ sera were collected and their binding activity to donor thymocytes was examined by flow cytometry. The production of IgM antibody against each donor was not observed (Fig. 5a,b). Interestingly, production of IgG type anti-donor antibody was observed only in a fully allogeneic combination (Fig. 5c,d). These results clearly indicate that MHC matching between the recipients and donors is effective for avoiding antibody-mediated rejection.

## Discussion

In this study, we established a murine tissue (skin) transplantation model of MHC homo grafting into hetero recipients. This is the first report describing the immune response in MHC homo-to-hetero transplantation, that is greatly implicated with iPSC-based transplantation. In this model, rejection was observed in most combinations between MHC homo donors and hetero recipients, known as MHC-matched but minor antigen-mismatched transplantation. Importantly, in some cases in our experiments (Fig. 1c), MHC homo grafts were rejected as early as those in MHC-fully-mismatched recipients. In fact, B6 skin grafts were lost as early as those derived from fully mismatched BALB/c mice following transplantation into MHC heterozygous C3129F1 recipients. These results indicate that B6 and C3129F1 mice have a substantial amount of minor antigen mismatches. In contrast, B6 skin grafts transplanted into C3B10F1 recipients were accepted for over 100 days. This is presumably because the B6 and B10 mouse strains were both derived from C57BL mice; genetic analysis has revealed that these strains are highly homologous^14^. In the practical transplantation of iPSC-derived cells or tissues, it is anticipated that there are not a few donor-recipient combination like that between B6 and C3129F1 strains. Therefore, it is important to establish strategies to regulate the immune rejection in iPSC-based, MHC-matched but minor antigen-mismatched transplantation.

In this study, vigorous CD4^+^ and CD8^+^ T cell reactions were observed in in vitro mixed lymphocyte reaction (MLR). Accordingly, T cell-depleted MHC hetero recipients administrated with anti-CD4 and anti-CD8 antibodies weekly before and after transplantation accepted the MHC homo skin graft for more than 6 weeks (our unpublished data). Therefore, T cells are important for the rejection in the minor antigen-mismatched graft rejection. However, immunosuppressants targeting T cell activation could not protect the grafts from rejection in this study. Further studies are needed to establish effective immunosuppressive regimens or other strategies for controlling the graft rejection. The murine transplantation model introduced in this paper would be helpful to develop such therapeutic maneuvers.

In this study, we found that MHC matching between donors and recipients was effective for reducing the production of de novo antibodies against donor antigens responsible for antibody-mediated rejection^10,15^. It is known that indirect pathway of alloantigen recognition mainly contributes to the development of post-transplant donor-specific antibodies (DSA), major of which recognize donor MHCs^13^. Although we have showed that indirect pathway was activated in the homo-to-hetero transplantation, MHC matching seems superior to prevent the production of DSA. It is well-known that once anti-donor antibodies emerge in the recipient's body, it is extremely difficult to control antibody production even if the recipients are administered immunosuppressants^13^. Therefore, MHC matching may contribute to long-term graft survival in iPSC-based transplantation. However, some tissue-specific antigens induce antibody production^16,17^. Our experiment was not able to detect DSA against skin-tissue specific antigens because we used thymocytes as donor cells. For these reasons, the involvement of anti-donor antibodies to rejection was not evaluated thoroughly in this study. Thus, although the risk of antibody-mediated rejection may be reduced, the rejection caused by DSA against minor antigens remains to be discussed in MHC-matched but minor antigen-mismatched transplantation. As described above, even when MHCs are matched, some donor-recipient combinations show graft rejection. Therefore, further studies are required to develop immuno-regulatory strategies for ensuring long-term graft survival in MHC homo-to-hetero transplantation.

## Materials and methods

### Mice

Male BALB/c (haplotype: H-2^d^), C57BL/6J (B6) (H-2^b^), CBA/N (H-2^k^), 129X1/Sv (H-2^b^), C57BL/10 (B10) (H-2^b^), and B6D2F1 (H-2^b/d^), and female C3H/He (H-2^k^) and B6 mice (5–8 weeks old) were purchased from Japan SLC, Inc. (Shizuoka, Japan). All animal procedures were approved by the Hokkaido University Animal Care Committee (Approval number: 17–0110) and the eperiments were performed in accordance with the Guide for the Care and Use of Laboratory Animals published by the National Institutes of Health.

### Skin transplantation

Recipient mice were anesthetized by intraperitoneal injection of a three-drug mixture of medetomidine (Domitor Nippon Zenyaku Kogyo Co., Ltd.), midazolam (midazolam injection, TEVA, Takeda Pharmaceutical Co., Ltd.), and butorphanol (Vetorphale, Meiji Seika). The dorsal side of the auricle skins from donor mice were transplanted into dorsal thoracic of the recipients. Recipient mice were wrapped with bandages for 7 days to protect skin grafts and warmed up until they moved freely as previously described^18^. To assess rejection, the graft diameter was measured. The day of graft rejection was determined as the day on which the graft diameter reached less than 30% of the initial diameter. Graft survival was calculated using the following formula: (total number of transplanted grafts − number of rejected grafts)/total number of transplanted grafts × 100. All the mice were euthanized by cervical dislocation at the end of experiments.

### Immunohistochemistry

The skin grafts were fixed in 4% paraformaldehyde and embedded in paraffin. The blocks were sliced into 5-µm sections and stained with anti-mouse CD3ε (A0452, Dako, Glostrup, Denmark) or anti-mouse Iba-1 (019-19741, Wako, Osaka, Japan) as the primary antibody, and then with anti-rabbit IgG-horse radish peroxidase (K4003, Dako) as a secondary antibody. Antibody binding was detected with a chromogenic substrate for horseradish peroxidase. The samples were counter stained with hematoxylin and eosin or Kernechtrot (Merck Millipore, Billerica, MA, USA).

### Mixed lymphocyte reaction

To assess the recipients’ T cell response evoked by the “direct pathway”, we performed a mixed lymphocyte reaction as follows. Splenic T cells were isolated from naïve C3129F1 mice using a Thy1.2^+^ cell isolation kit (Miltenyi Biotec, Gladbach Bergisch, Germany) and used as responder cells. Bone-marrow-derived dendritic cells from BALB/c, B6, CBA/N, or C3129F1 mice were used as stimulator cells. Carboxyfluorescein diacetate succinimidyl ester (CFSE, Dojindo Laboratories, Kumamoto, Japan)-labeled responder cells (2 × 10^5^) were co-cultured with stimulator cells (1 × 10^4^) in 96-well round-bottomed culture plates in RPMI-1640 high-glucose supplemented with 10% fetal bovine serum, 0.1 mM non-essential amino acids, 1 mM sodium pyruvate, 50 µM 2-mercaptoethanol, 100 U/mL penicillin, and 100 µg/mL of streptomycin (all from Life Technologies, Carlsbad, CA, USA). On day 4 of the culture, T cell proliferation was assessed by measuring the reduction of CFSE fluorescence. To assess the recipients' T cell response evoked by an “indirect pathway”, we performed a mixed lymphocyte reaction as follows. Recipient mice splenocytes were depleted of CD19^+^ cells using a CD19 cell isolation kit (Miltenyi Biotech) and used as responder cells. As stimulator cells, 30 Gy-irradiated whole splenocytes were used. Responder cells (2 × 10^5^) were co-cultured with stimulator cells (1 × 10^5^) in 96-well round-bottomed culture plates. On day 4 of culture, T cell proliferation was assessed by measuring the reduction in CFSE fluorescence by flow cytometry.

### Flow cytometry and antibodies

Flow cytometry was performed using an FC500 instrument (Beckman Coulter, Brea, CA, USA), FACSCanto II (BD Biosciences, Franklin Lakes, NJ, USA), FACSAria II (BD Biosciences), or FACSCelesta (BD Biosciences), and the data were analyzed by FlowJo software V7.6.5 (purchased from Tree Star, Ashland, OR, USA). Phycoerythrin (PE)-anti-CD4 (RM4-5), allophycocyanin (APC)-anti-CD8α (53–6.7), biotin-anti-CD3ε (145-2C-11), APC-cyanin 7 (Cy7)-anti-TCRβ (H57-597), biotin-anti-CD11b (M1/70), biotin-anti-CD11c (N418), streptavidin-APC-Cy7, and streptavidin-brilliant violet 510 were purchased from Biolegend (San Diego, CA, USA). For analysis, live cells were gated based on forward and side scatter as well as a lack of propidium iodide (PI) or 4′,6-diamidino-2-phenylindole (DAPI) uptake.

### Measurement of alloantibody production

In vivo antibody production was analyzed as previously described^18^. Briefly, 40–69 days after skin graft transplantation, the sera were harvested from C3129F1 recipient mice by blood collection. To detect antibodies against the donor antigen in the sera, thymocytes from BALB/c, B6, CBA/N, or C3129F1 mice were Fc receptor-blocked, and then incubated with the sera. After washout of unbound antibodies, the cells were further incubated with Alexa fluor-488-conjugated goat anti-mouse IgG antibody (Invitrogen, Carlsbad, CA, USA) or PE-conjugated anti-mouse IgM antibody (Biolegend), and then analyzed with an FC500 instrument and FlowJo software.

### Immunosuppressants

Tacrolimus was obtained from Astellas (Tokyo, Japan). Tacrolimus was diluted with saline. Recipient mice were administered daily intraperitoneal injections of 0.5 or 2.0 mg/kg tacrolimus starting on the day of transplantation. Rapamycin was obtained from LC Laboratories (Woburn, MA, USA). Rapamycin was dissolved in dimethyl sulfoxide (Sigma-Aldrich, St. Louis, MO, USA) and diluted with phosphate-buffered saline. Recipient mice were administered intraperitoneal injections of 1.0 mg/kg rapamycin each day starting on the day of transplantation.

### Statistics

Statistical data were analyzed with JMP software (JMP Version 13.1.0, SAS Institute, Inc., Cary, NC, USA; JMP license was distributed by Hokkaido University Graduate School of Medicine.). Kaplan–Meier survival curves were analyzed using the log-rank test or Tukey’s honestly significant difference (HSD) test. *p* values less than 0.05 were considered to indicate statistically significant results.

## Data Availability

The datasets generated during and analyzed during the current study are available from the corresponding author on reasonable request.
